# The Impact of Low-Level Laser Therapy on Spasticity in Children with Spastic Cerebral Palsy: A Systematic Review

**DOI:** 10.3390/brainsci14121179

**Published:** 2024-11-25

**Authors:** Amalio Jiménez, Frederick R. Carrick, Norman Hoffman, Monèm Jemni

**Affiliations:** 1 The Carrick Institute, Cape Canaveral, FL 32920, USA; drfrcarrick@post.harvard.edu (F.R.C.); drnorm@hoffmanwellness.com (N.H.); monemj@hotmail.com (M.J.); 2 Centre for Mental Health Research in Association with the University of Cambridge, Cambridge CB2 1TN, UK; 3 College of Medicine, University of Central Florida, Orlando, FL 32827, USA; 4 Burnett School of Biomedical Science, University of Central Florida, Orlando, FL 32827, USA; 5 Department of Health Professions Education, MGH Institute for Health Professions, Boston, MA 02129, USA; 6 Faculty of Physical Education, Ningbo University, Ningbo 315000, China

**Keywords:** low-level laser therapy, spasticity, cerebral palsy, children

## Abstract

Context: Spastic cerebral palsy (SCP) is a condition characterized by muscle stiffness and involuntary movements, which greatly affect movement abilities and overall well-being. Low-level laser therapy (LLLT) has emerged as a treatment option for managing spasticity, though the current evidence varies. Objective: This systematic review seeks to assess the efficacy of LLLT on spasticity in children with cerebral palsy. We hope it will pinpoint areas where more research is needed and suggest directions for future studies. Method: A search of the literature was performed across databases, such as PubMed, Google Scholar, Scopus, and Elicit. The search utilized keywords and the Medical Subject Headings (MeSH) terms. Only studies conducted in English that focused on children with cerebral palsy (CP) and explored the effects of LLLT on spasticity were considered. The quality of the selected studies was evaluated using assessment tools. Results: The search identified 534 references, out of which eight studies met the screening criteria for inclusion. All cited papers indicated reductions in spasticity with further mention of reduced pain and greater muscle strength by some authors. Conclusions: This review indicates that LLLT shows promise in decreasing spasticity in children with cerebral palsy. Nevertheless, a lack of treatment parameters, heterogeneity in research methods, and a lack of objective outcome measures weaken the results. This review underscores the importance of standardized procedures and carefully planned randomized controlled trials to establish conclusive findings on the effectiveness of LLLT in this population.

## 1. Introduction

Cerebral palsy is a group of permanent motor development disorders and body postures that causes limited activity [1]. The condition is correlated to damage or lesions of the central nervous system during the prenatal, perinatal, and postnatal periods, when the central nervous system has not been well developed [2]. Rather than a single entity, CP is an umbrella term used to define a group of non-progressive neurological disorders of movements and postures and is one of the leading causes of childhood motor disability [3]. Research reveals that many children with cerebral palsy develop spasticity [4]. Of the three types of CP (spastic, dyskinetic, and ataxic), spastic cerebral palsy is the most common form of CP, characterized by muscle tightness, stiffness, and involuntary muscle movements [4,5]. Spasticity, which is distinguished by changes in the intensity and speed of movement, is a form of hypertonia. Spasticity impacts mobility and quality of life in most cases [4]. The search for treatments to address spasticity in children with spastic cerebral palsy has led to an interest in alternative therapeutic methods [6]. One emerging approach is low-level laser therapy (LLLT), a treatment that stimulates function and enhances tissue healing [7]. Unlike rigidity, such as that which occurs in Parkinson’s disease, spasticity often asymmetrically affects opposing muscle groups, as it arises from damage to pyramidal tracts [8]. Supporting a child with spastic cerebral palsy to maximize their full abilities is best achieved using a multidisciplinary approach, commencing treatment as soon as possible. This sets the stage for the consideration of low-level laser therapy (LLLT) as a way to further lessen muscle stiffness in children with CP [9].

LLLT using low-power lasers or light-emitting diodes has gained attention as a treatment for spasticity in children with spastic cerebral palsy. This novel approach is being studied for its ability to alter cellular activity by such means as photochemistry, as light energy is absorbed by cellular chromophores [10,11].

Furthermore, Yang et al. (2021) conducted a study reviewing the use of photobiomodulation in individuals with nervous system conditions [7]. Photobiomodulation, which involves using near-infrared light, has been found to stimulate the growth of neurons and trigger anti-cell death, anti-inflammatory, and antioxidant reactions. Research has shown that photobiomodulation offers benefits for conditions such as neurological disorders, peripheral nerve damage, pain management, and wound recovery [12].

Low-level laser therapy (LLLT), also known as photobiomodulation, is a therapeutic approach that employs low-intensity light sources, such as specialized lasers or light-emitting diodes, to promote tissue repair, alleviate discomfort, and mitigate inflammatory responses in the body [13]. LLLT involves applying light of specific wavelengths, typically in the red or near-infrared spectrum, to the skin or affected tissues. LLLT is thought to work through several mechanisms. Light is absorbed by photoacceptors in cells, particularly cytochrome c oxidase in the mitochondria, stimulating ATP production and increasing cellular energy [14]. The therapy can cause vasodilation, improving circulation in the treated area. It may also reduce the production of inflammatory mediators and increase anti-inflammatory factors, while potentially stimulating the synthesis of growth-promoting factors that facilitate tissue healing, and renewal may be stimulated by this therapy [15]. In the context of spasticity management, LLLT has garnered scientific interest as a possible intervention, although its efficacy remains under scrutiny [16]. Hypothesized mechanisms of action include muscle tone reduction through enhanced circulation and decreased inflammation in muscular and adjacent tissues, potentially fostering the relaxation of hypertonic muscles [17]. Certain research indicates that LLLT might modulate the release of neurotransmitters crucial to muscle contraction and relaxation processes. The therapy’s ability to boost blood flow could potentially enhance oxygen delivery to affected muscles, possibly leading to improved muscular function [18]. Additionally, by diminishing spasticity-associated pain, LLLT may indirectly contribute to reducing muscle tension, offering a multi-faceted approach to addressing this complex condition [19]. It is important to understand what LLLT might achieve for spasticity. The therapy could temporarily relieve muscle tightness, reduce pain associated with spasticity, and improve local circulation in treated areas [16,20].

While LLLT may not improve functionality directly, it shows promise in promoting tissue recovery and managing conditions and pain relief [21]. In one example, LLLT was administered to the masseter and anterior temporalis muscles in children with spastic CP in six sessions spanning six weeks. Following the completion of these treatments, subjects undergoing LLLT showed continuous improvement [22]. Nevertheless, their progress reverted to baseline levels by the end of the sixth week. These results offer the possibility that temporarily decreased spasticity could offer therapists an opportunity to conduct more impactful stretching exercises, potentially leading to greater outcomes.

Numerous research studies have investigated how LLLT impacts spasticity in children with spastic cerebral palsy [13]. The consensus of most of these studies is that there is a need for an overview of the existing evidence. This systematic review seeks to assess the efficacy of LLLT on spasticity in children with cerebral palsy. It pinpoints areas where more research is needed and suggests directions for future studies.

## 2. Methods

### 2.1. Search Strategy

A literature search was conducted across multiple electronic databases, including PubMed, Google Scholar, Scopus, and Elicit, using a combination of relevant keywords and (MeSH) terms related to “Low-Level Laser Therapy”, “spastic cerebral palsy”, and “spasticity”. Boolean operators (AND, OR) and synonyms were utilized to refine the search and capture diverse studies. These studies employed research methodologies like randomized controlled trials, cohort studies, and case–control studies.

### 2.2. Inclusion and Exclusion Criteria

The criteria for selection included research articles from journals that examined individuals and children diagnosed with cerebral palsy and assessed the impact of LLLT on spasticity. We looked at a range of study types for inclusion, such as randomized controlled trials (RCTs), cohort studies, case–control studies, and observational studies. We adjusted the time frame to the past 10 years (2014–2024). Only studies that were published in English were considered.

Studies not primarily centered on LLLT as an intervention, those with insufficient data or unclear methodology, those involving participants outside the pediatric age group, and studies not reporting outcomes related explicitly to spasticity in cerebral palsy were excluded. Non-English language publications were also excluded.

One study enrolled 40 children with hemiplegic spastic cerebral palsy, ranging from 1 to 4 years old (mean age: 3 years and 2 months) [23]. Another study included 40 children aged 5–18 years old, all diagnosed with spastic diplegia [16].

In 1 study, the laser therapy was applied using a gridding technique on the back of the thigh and leg, with the probe in continuous mode at ninety degrees, with light pressure on the skin for 40 s per square centimeter of the treatment area [24]. The duration of the laser therapy treatment in another study was 1 month, with significant improvements noted in various parameters such as pain levels and lactate levels in the blood [16].

### 2.3. Study Selection and Data Extraction

The research selection process adhered to the guidelines outlined in the Preferred Reporting Items for Systematic Reviews and Meta Analyses (PRISMA) [25]. Following the removal of duplicate entries, the contributing authors in this work, FRC, NH, and MJ, and I proceeded to examine the titles and abstracts of the identified studies according to the inclusion and exclusion criteria. Subsequently, full-text articles were evaluated for suitability. Any discrepancies were addressed through conversation and mutual agreement.

The data were systematically extracted using a standardized data collection form. 

### 2.4. Study Selection

The initial database search retrieved 534 references. After adjusting the time frame to the past 10 years (2014–2024), 287 studies were analyzed by their titles and abstracts, and 247 were excluded. A total of 20 studies were read in full, among which 11 were excluded due to being unrelated to children or cerebral palsy.

**Figure 1:**
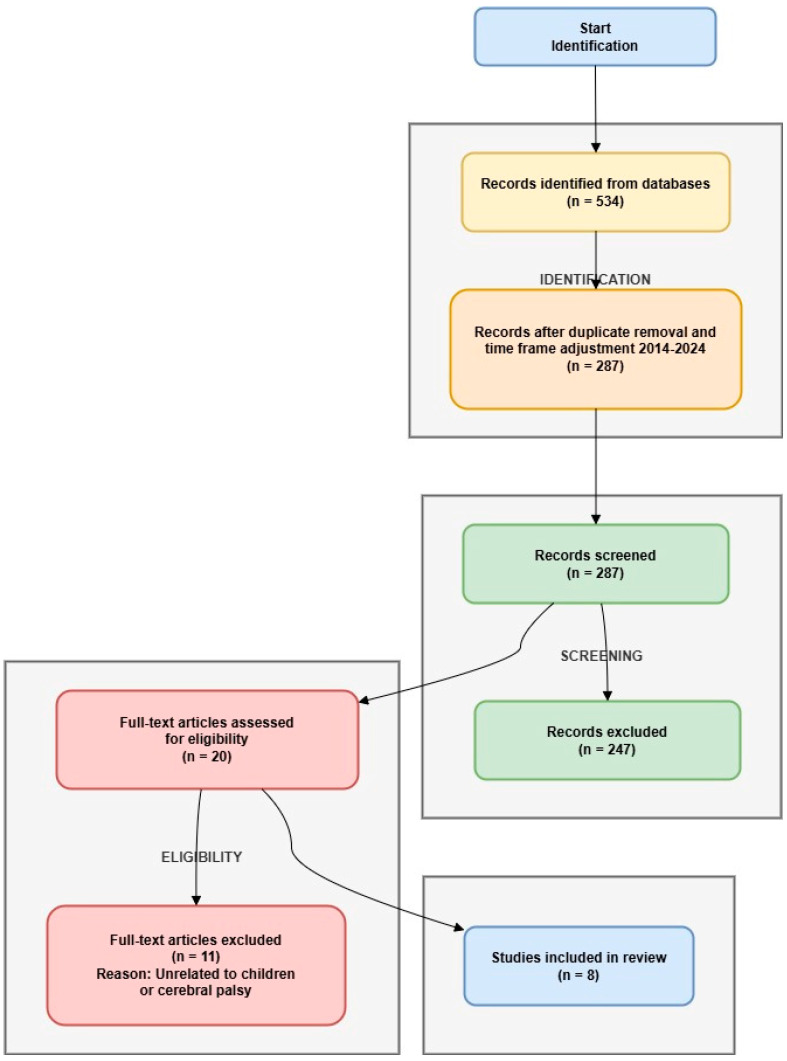
PRISMA flow diagram of the study selection process.

In total, 8 articles were considered eligible for this systematic review. The selection cycle is in accordance with the Preferred Reporting Items for Systematic Reviews and Meta-Analyses (PRISMA) guidelines and is represented as a flowchart in Figure 1.

The selected randomized controlled trials were assessed for their quality by utilizing the Cochrane Risk of Bias Tool, which scrutinizes aspects of bias in RCTs, including the creation of random sequences and the concealment of allocations, as well as the blinding of participants and staff along with outcome assessments and incomplete outcome data, among others. 

Due to the variations in research designs, interventions, and outcome measures among the included studies, a meta-analysis was not considered appropriate. Instead, a qualitative synthesis of the findings was conducted using a storytelling method, systematically summarizing the results, and drawing conclusions from individual studies.

## 3. Results

Table 1 presents the extracted information, including study characteristics (author, publication year, and study design), participant demographics, intervention details (low-level laser therapy parameters, dosage, and duration), outcome measures, and main findings.

The literature on the effects of low-level laser therapy on spasticity in children with spastic cerebral palsy (SCP) is relatively sparse, despite a comprehensive search. Only a limited subset met the inclusion criteria. It is noteworthy that all cited investigations into the efficacy of LLLT on examinations of spasticity utilized a therapeutic intervention at the level of the muscle in human subjects, although several were performed in the context of laser acupuncture.

The systematic review process yielded eight studies that met our inclusion criteria, providing a diverse yet interconnected body of evidence on the effects of low-level laser therapy (LLLT) in managing spasticity among children with CP.

Abdelhalim’s (2023) study set the stage by demonstrating LLLT’s potential in reducing muscle fatigue in children with spastic diplegia. This finding is particularly significant as it addresses a key component of motor function in CP. Building on this, Dabbous et al. (2016) explored LLLT’s role as an adjunctive therapy, revealing its beneficial effects when combined with conventional treatments. This multi-modal approach opens up new possibilities for comprehensive CP management [16].

Eitedal et al. (2021) furthered the investigation into LLLT’s impact on specific muscle groups, focusing on lower limb muscles. Their positive findings regarding increased muscle power complement Abdelhalim’s work on reduced fatigue, suggesting the multi-faceted effect of LLLT on muscle function in CP [26].

Putri et al. (2020) shifted the focus to spasticity, demonstrating LLLT’s potential to reduce this key symptom of CP. This study provides a crucial link between the observed muscle function improvements and spasticity’s clinical manifestation, making it highly relevant to our practice [27]. Ragab (2021) then narrowed the focus to specific muscle groups—hamstrings and calves—commonly affected in CP. Their findings of reduced muscle tightness and spasticity in these areas offer valuable insights for targeted LLLT application, further enhancing the clinical relevance of our work [24].

Santos et al.’s (2017, 2016) work has brought to light an often-overlooked aspect of CP: oral health and masticatory function [22]. Their studies on masseter muscle thickness and spasticity have revealed the potential of LLLT in areas beyond limb function, suggesting a broader scope for this therapy [28]. This intriguing potential of LLLT could significantly enhance the quality of life for children with CP, sparking our curiosity about its application in CP treatment.

To truly grasp the impact of LLLT in managing spasticity in individuals with cerebral palsy, it is essential to have a deep understanding of the complexity of this condition. Nevertheless, the promise of LLLT in enhancing spasticity management and enhancing functionality in children with cerebral palsy paints an optimistic picture for what lies ahead [9].

Collectively, these studies paint a promising picture of LLLT’s potential in CP management. They suggest benefits ranging from reduced muscle fatigue and increased power to decreased spasticity across various muscle groups. However, the variability in study designs, LLLT parameters, and outcome measures underscores the need for further standardized research to solidify these findings.

## 4. Discussion

The main goal when treating spasticity in children with spastic cerebral palsy (SCP) is to improve their everyday abilities. Additionally, it is important to prevent muscle stiffness and contractions through treatment. This review aims to summarize the existing evidence on how LLLT affects spasticity in children with this condition. The results indicate that LLLT could be a method for reducing spasticity and enhancing muscle tone and function in these individuals. However, due to the variations in research methods and outcomes it is essential to interpret the findings and stress the significance of conducting planned larger studies.

Multiple studies reported promising outcomes, with LLLT demonstrating beneficial effects on spasticity, muscle tone, and functional abilities [22,23,27]. These findings are supported by the proposed mechanisms of LLLT, which involve stimulating biological processes such as tissue repair, reducing inflammation, and modulating neuronal function [7,10]. However, other studies showed more modest or inconclusive results, indicating that further research is needed to establish the optimal parameters and protocols for LLLT in managing spasticity in children with SCP [16,27,28].

LLLT has been proposed as a treatment for reducing spasticity in children with SCP. Nevertheless, there are conflicting opinions on its effectiveness. While specific studies and sources back the effects of LLLT on spasticity, some researchers offer other viewpoints. Putri et al. suggest that the scientific proof supporting the effectiveness of LLLT for spasticity in children with CP is limited [27]; meanwhile, Santos et al. present evidence of the advantages of using LLLT in treating spasticity. Also, outcomes can vary significantly among patients, with some showing minimal improvements [22]. The discussion around the long-term effects of LLLT on spasticity in children with CP is ongoing, with some studies indicating that the benefits may not be sustained over time [30]. Although some sources endorse the impact of low-level laser therapy on spasticity in children with SCP, differing perspectives and evidence raise doubts about its efficacy and lasting benefits. Further exploration through research and clinical trials might be needed to understand how effective LLLT is in this specific scenario.

Each study investigated in this review used different LLLT settings and outcome measures, which may explain the variation in results [9]. Factors like the type of light, dosage, and duration of LLLT, along with which muscles were targeted and how severe the spasticity was, might affect how well the treatment works [31]. The quality of the studies also varied. Some had bias risks because of things like sample sizes, no randomization and potential variables that could confuse the results [32]. These limitations show why we need to design studies on a scale with proper blinding and control groups to obtain stronger proof on how effective LLLT is in reducing spasticity in kids with SCP [33]. While some such studies have been found, there are gaps in the research. Most of the focus has been on the effects of LLLT on spasticity without information on its potential long-term benefits. It is also important to consider publication bias, where positive results might be highlighted more than other ones, due to reasons like perceived significance or novelty. By addressing these gaps in research, we can gain an understanding of how effective and lasting LLLT is as a treatment for spasticity.

In the future it would be beneficial for researchers to overcome these limitations by conducting well-structured randomized controlled trials (RCTs), with low-level laser therapy (LLLT) protocols and extended follow-up periods. Comparative studies that assess the effectiveness of LLLT compared to established therapies or in conjunction with other treatments could yield valuable insights [34,35]. Low-level laser therapy (LLLT) is a method for treating spasticity, and studies comparing its effectiveness with other treatments or in combination with other therapies could offer valuable insights [20,36].

On the other hand, LLLT is a noninvasive method that uses low-intensity light energy to stimulate cellular processes and support tissue healing [17,37]. The proposed mechanisms by which LLLT reduces spasticity include the regulation of oxidative stress responses, enhancement of ATP production and cellular metabolism, promotion of tissue repair and regeneration, and modulation of neurotransmitter release at synapses [14]. Unlike medications, LLLT does not pose the risk of side effects. Additionally, unlike botulinum toxin injections it is noninvasive [38] and could potentially offer longer-lasting effects [39]. However, researchers are still exploring the depth of penetration and the specific mechanisms through which LLLT impacts spasticity [40]. Comparative studies could evaluate how effective LLLT is compared to established therapies like botulinum toxin injections and investigate any effects when combining LLLT with other treatments [41,42]. These studies may also investigate how lasting the effects of LLLT are in contrast to the relief from botulinum toxin injections or potential reductions in oral medication dosages [43].

Physical and occupational therapy are essential for managing spasticity through methods such as stretching, range-of-motion exercises, and functional training. These therapies focus on enhancing mobility, preventing contractures, improving function, and increasing independence [44,45]. Orthotic devices like ankle foot orthoses or splints can also be used to counteract muscle tone and positioning [46].

Surgery like dorsal rhizotomy or orthopedic procedures such as tendon releases or muscle lengthening surgeries may be considered for spasticity that does not improve with conservative treatments. These procedures involve changes to the system and are usually chosen for specific cases [47]. Other therapies like acupuncture, massage therapy, and whole-body vibration therapy are being studied as treatments for spasticity [48]. More research is needed to understand how effective they are [49,50].

Recently, shock wave therapy has shown positive results as an alternative for treating spasticity. It also demonstrates potential use for pain reduction, motor function improvement, reduced motor impairment, and improved functional independence [51].

Comparative studies could compare how well LLLT works compared to these therapies alone or in combination with them. These studies could look at how the effects of LLLT last, if it works better when combined with treatments, and if it can reduce the need for surgery or lower doses of medications. Because LLLT is noninvasive and does not involve medication, it is worth exploring. Additionally, research focusing on patient-reported outcomes, such as capabilities and quality of life, may offer a comprehensive understanding of how LLLT impacts the overall well-being of children with SCP [9,17,37,39].

### Potential Biases and Limitations of This Review

In conducting this review, we encountered several potential biases and limitations that warrant careful consideration when interpreting our findings. These factors, if not addressed, could significantly impact the generalizability and overall conclusions of our study, underscoring the gravity of the situation.

One significant limitation stems from our decision to include only English-language studies. This approach may have excluded valuable research published in other languages, potentially skewing our findings toward work conducted in English-speaking countries or regions. Consequently, our review may only partially represent the global research landscape on LLLT in cerebral palsy.

Publication bias presents another challenge, as there is a well-documented tendency for positive results to be published more readily than negative or null findings. This can lead to the ‘file drawer problem’, where unpublished negative studies could exist but are not included in the review. If these studies were included, they could potentially alter our conclusions about LLLT efficacy in managing spasticity in children with cerebral palsy.

While our selection criteria were designed to ensure the quality of included studies, we may have inadvertently introduced bias. Despite our systematic approach, we may have omitted relevant studies, particularly those with negative findings that needed to meet our quality thresholds. This selection bias could skew our overall conclusions.

Interpretation bias is another factor to consider. Our analysis of the studies’ results may be influenced by our expectations or prevailing opinions in the field, potentially leading to an unconscious emphasis on findings that align with preconceived notions about LLLT’s effectiveness.

The limited number of studies meeting our criteria—only eight in total—presents a significant limitation. This small sample size may not fully represent the true effects of LLLT in cerebral palsy and could lead to the over- or underestimation of its impact. Therefore, there is an urgent need for more comprehensive studies. Furthermore, the heterogeneity among these studies regarding methodologies, LLLT parameters, outcome measures, and patient characteristics makes it challenging to draw definitive conclusions. It may limit the generalizability of our findings.

Most of these studies focused on short-term outcomes, leaving a gap in our understanding of the long-term effects of LLLT. This lack of long-term follow-up data limits our ability to assess the durability of LLLT’s effects and its long-term safety profile in children with cerebral palsy.

We must also consider the potential for reporting bias in the original studies. Our review relies on the information provided in published papers, and if the original authors selectively reported their outcomes or incompletely disclosed their methodologies, this could impact the accuracy of our review. For instance, if studies with positive results were more likely to be published, our review could overestimate the effectiveness of LLLT.

Another area for improvement is our reliance on study-level data rather than individual patient data. This restricts our ability to conduct more nuanced analyses that could reveal important subgroup effects or individual-level predictors of LLLT response. For example, we may miss out on understanding how LLLT affects different age groups or severity levels of cerebral palsy.

Our search strategy may have captured only some relevant grey literature, such as conference proceedings, unpublished theses, or government reports. This could result in the omission of potentially valuable data.

It is crucial to be aware that a single-reviewer approach, including study selection and data extraction, carries the potential for individual bias. This understanding can help us be more cautious and aware of limitations. A multi-reviewer approach with inter-rater reliability measures would have significantly strengthened the robustness of the review process.

It is of utmost importance to explicitly consider the funding sources of the included studies. Industry-funded studies, in particular, might be more likely to report positive outcomes, which could significantly influence our overall conclusions if not properly accounted for.

These potential biases and limitations underscore the need for us to interpret our findings carefully. They also highlight the importance of our commitment to further rigorous research in this field. Large-scale, well-designed, and randomized controlled trials with standardized protocols and long-term follow-up are the way forward.

Future reviews could address these limitations by including studies in multiple languages, conducting a more comprehensive search for unpublished data, using more inclusive selection criteria while maintaining quality standards, employing multiple independent reviewers, conducting sensitivity analyses to assess the impact of potential biases, focusing on patient-level data when available, and explicitly considering and analyzing the impact of funding sources on study outcomes.

Recognizing these constraints is crucial as we strive to offer a basis for understanding our discoveries and directing research endeavors in this critical realm of pediatric healthcare provision. Our study acts as a driving force for promoting more thorough inquiries while underscoring the importance of enhancements in pediatric care.

## 5. Limitations and Gaps in Evidence

While the studies included offered insights into how LLLT could impact children with CP dealing with spasticity, there are some limitations and gaps in the evidence. The methodological quality of the included studies varied. Most RCTs were considered to have a low or moderate risk of bias. Given that the studies are primarily randomized controlled trials (RCTs), the Cochrane Risk of Bias Tool for RCTs is the most appropriate tool. This tool assesses several domains of potential bias in RCTs.

In contrast, some observational studies had a higher risk of bias due to lack of randomization, inadequate blinding, and potential confounding variables. Some studies lacked sample sizes, which may have affected their strength and applicability. Another challenge was the variability among studies in terms of LLLT parameters like wavelength, dosage, and duration, as differences in treatment protocols and outcome measures were used, making it difficult to compare results. Additionally, most studies focused on short-term effects providing information on the short-term impact of LLLT on spasticity. Finally, there might be a chance of publication bias where studies with insignificant results were less likely to be published, potentially influencing the evidence available.

## 6. Conclusions

This systematic review provides an overview of the current evidence on the effects of LLLT on spasticity in children with spastic cerebral palsy. While the findings suggest that LLLT holds potential as a therapeutic approach, the variations in study methodologies and outcome measures highlight the need for caution in interpreting the results. Well-designed, larger-scale RCTs with standardized protocols and long-term follow-up are necessary to establish the efficacy and optimal parameters of LLLT in managing spasticity in this population.

Future research should focus on addressing the identified gaps in the literature, including conducting comparative studies, assessing long-term outcomes, and evaluating patient-reported outcomes to provide a comprehensive understanding of the impact of LLLT on the overall well-being of children with spastic cerebral palsy. By addressing these limitations and conducting high-quality research, the potential of LLLT in managing spasticity in children with spastic cerebral palsy can be more accurately assessed, ultimately informing clinical practice and improving the quality of life for these individuals.

Based on the research available, LLLT treatment seems to show promise as an option for kids with spastic cerebral palsy, by reducing spasticity and enhancing muscle function. The fact that it is noninvasive and has a good safety record makes it an appealing substitute for drugs or surgery. However, differences in how studies are conducted, and their results, indicate that using LLLT treatment needs to be thought out. Doctors should view LLLT treatment with optimism, as one part of a complete treatment plan, rather than solely relying on it. Though low-level laser therapy (LLLT) comes with risks and numerous advantages, it is crucial to tailor its application according to the requirements of each patient, while also combining it with existing treatments and monitoring its efficacy closely. As studies advance, there is the possibility of procedures becoming more prevalent, thereby enabling an assured and extensive use of LLLT on children with cerebral palsy. This prospect of development and enhancement nurtures our hope for the future of LLLT usage. In the end, even though the existing findings back up the idea of investigating LLLT as a viable treatment choice, they highlight the importance of conducting comprehensive studies to confirm its effectiveness in addressing spasticity in kids with cerebral palsy. This emphasizes how crucial it is for healthcare providers to keep up to date on the research in this changing domain, to ensure optimal care for their CP patients.

## Figures and Tables

**Table 1:** Summary of studies on laser therapy for spasticity in children with spastic cerebral palsy.

Authors	Objective	Key Findings	Theoretical Framework	Method (Application Site, Frequency, Duration)	Gaps
Abdelhalim, 2023 [16]	Effect of low-level laser therapy on quadriceps and foot muscle fatigue in children with spastic diplegia.	Low-level laser therapy (LLLT) decreased quadriceps and foot muscle fatigue in children with spastic diplegia.	The study is grounded on the application of low-level laser therapy and its potential impact on muscle fatigue in spastic diplegia.	Randomized controlled study conducted on children with spastic diplegia, measuring the effects of LLLT on quadriceps and foot muscle fatigue. LLLT on quadriceps and feet, 6 sessions over 6 weeks. Utilizing the Ashworth Scale, and EMG as a measuring tool.	Further research could explore the long-term effects and potential drawbacks of LLLT, as well as its effectiveness in comparison to other therapies.
Dabbous, 2016 [23]	Laser acupuncture as an adjunctive therapy for spastic cerebral palsy in children.	Laser acupuncture serves as a beneficial adjunctive therapy for spastic cerebral palsy in children.	The paper discusses the role of laser acupuncture as a supplementary treatment method for spastic cerebral palsy.	This cohort study employs laser acupuncture as an adjunctive therapy alongside conventional treatments for spastic cerebral palsy in children. Laser acupuncture applied at specific acupoints for 4 weeks. Modified Ashworth Scale, and visual analog scale utilized (VAS).	More studies could focus on the comparative effectiveness of laser acupuncture versus other adjunctive therapies for spastic cerebral palsy.
Eitedal, 2021 [26]	Effect of laser acupuncture on the power of lower limb muscles in children with spastic cerebral palsy.	Laser acupuncture positively impacts the power of lower limb muscles in children with spastic cerebral palsy.	The study investigates the influence of laser acupuncture on the muscular power of lower limbs in children with spastic cerebral palsy.	Research focuses on evaluating the effect of laser acupuncture on lower limb muscle power in children with spastic cerebral palsy. This is a case–control study. Laser acupuncture applied to lower limbs over 10 sessions, then Manual Muscle Testing (MMT) was utilized as a measuring tool.	Future studies could explore the mechanisms underlying the observed effects of laser acupuncture on muscular power and potential variations based on severity or subtype of cerebral palsy.
Putri, 2020 [27]	Effect of laser acupuncture on spasticity in children with spastic cerebral palsy.	Laser acupuncture demonstrates potential in reducing spasticity in children with spastic cerebral palsy.	The research assesses the efficacy of laser acupuncture in mitigating spasticity among children diagnosed with spastic cerebral palsy.	This RCT study examines the impact of laser acupuncture on spasticity levels in children with spastic cerebral palsy through a clinical trial. Laser acupuncture applied bi-weekly, over 5 weeks. The measuring tool was a Modified Tardieu Scale.	Further investigations could explore the durability of effects post-treatment and the optimal frequency and duration of laser acupuncture sessions for managing spasticity in this population.
Ragab, 2021 [24]	Effect of low-level laser therapy on hamstring muscle tightness and calf muscle spasticity in children with cerebral palsy.	Low-level laser therapy (LLLT) reduces hamstring muscle tightness and calf muscle spasticity in children with cerebral palsy.	The study explores the impact of LLLT on muscular tightness and spasticity, specifically targeting hamstring and calf muscles in children with cerebral palsy.	This RCT type of research employs LLLT as a therapeutic intervention to alleviate muscular tightness and spasticity in hamstring and calf muscles among children with cerebral palsy. LLLT applied on hamstrings and calves for 8 sessions and then the Ashworth Scale was utilized to measure results.	Future research could delve into the optimal parameters of LLLT application for addressing muscular tightness and spasticity in different muscle groups and severity levels of cerebral palsy.
Santos, 2017 [28]	Efficacy of photobiomodulation therapy on masseter thickness and oral-health-related quality of life in children with spastic cerebral palsy.	Photobiomodulation therapy (PBMT) shows efficacy in improving masseter muscle thickness and oral-health-related quality of life in children with spastic cerebral palsy.	The study examines the effectiveness of PBMT in enhancing masseter muscle thickness and oral-health-related quality of life in children diagnosed with spastic cerebral palsy.	This is an observational study. This research utilizes PBMT to evaluate its impact on masseter muscle thickness and oral-health-related quality of life among children with spastic cerebral palsy. PBMT applied on masseter muscle for 4 weeks, then the masseter thickness was measured by Ultrasound.	Future studies could explore the broader effects of PBMT on other facial muscles and functional aspects of oral health, as well as its sustainability over extended periods.
Santos, 2016 [22]	Evaluation of low-level laser therapy in the treatment of masticatory muscle spasticity in children with cerebral palsy.	Low-level laser therapy (LLLT) demonstrates effectiveness in treating masticatory muscle spasticity in children with cerebral palsy.	The study assesses the efficacy of LLLT as a therapeutic approach for managing masticatory muscle spasticity in children diagnosed with cerebral palsy.	This is a case–control type of study. The investigation employs LLLT as an intervention method to alleviate masticatory muscle spasticity in children with cerebral palsy, focusing on its treatment effectiveness. LLLT applied twice weekly for 6 weeks. Then a clinical spasticity grading scale was the measuring tool.	Further research could delve into the optimal dosage and duration of LLLT sessions for managing masticatory muscle spasticity, as well as its comparative effectiveness against other treatment modalities.
Sadowska, 2020 [29]	Cerebral palsy: current opinions on definition, epidemiology, risk factors, classification, and treatment options.	Provides current opinions and insights on the definition, epidemiology, risk factors, classification, and treatment options for cerebral palsy.	The paper presents an overview of current opinions and perspectives on various aspects related to cerebral palsy, including its definition, epidemiology, risk factors, classification, and treatment options.	Review article synthesizing the existing literature and expert opinions on cerebral palsy, incorporating findings from various studies and clinical experiences. It is an important review of LLLT and other therapies.	Future research could explore emerging treatment options and novel therapeutic approaches, as well as address ongoing challenges in defining and classifying cerebral palsy.
